# Modulating the tumor microenvironment: The role of traditional Chinese medicine in improving lung cancer treatment

**DOI:** 10.1515/biol-2025-1100

**Published:** 2025-05-20

**Authors:** Geling Teng, Min Zhang, Yuling Pan, Sajad Karampoor, Rasoul Mirzaei

**Affiliations:** Department of Respiratory and Critical Care Medicine, Shandong Public Health Clinical Center, Shandong University, Jinan, 250013, China; Department of Respiratory and Critical Care Medicine, Shandong Provincial Hospital Affiliated to Shandong First Medical University, Jinan, 250021, China; School of Medicine, Shandong University of Traditional Chinese Medicine, Jinan, 250355, China; Gastrointestinal and Liver Diseases Research Center, Iran University of Medical Sciences, Tehran, Iran; Novin-sin Biotechnology Inc., Tehran, Iran

**Keywords:** lung cancer, TME, TCM, immunomodulation, apoptosis, metastasis: Side effect

## Abstract

The holistic approach of traditional Chinese medicine (TCM) has been increasingly being focused on as a potential adjuvant to conventional lung cancer therapies in an attempt at modulating the tumor microenvironment (TME). Covering a diverse range of herbal medicine, acupuncture, and dietary therapy, TCM brings a unique perspective to influencing the TME. Importantly, the study has found the effects of specific TCM compounds, such as cantharidin, boehmenan, shikonin, and salidroside, on lung cancer in the TME. These compounds interact intricately with key apoptotic regulators, oxidative stress pathways, and inflammation-related mechanisms, suggesting their potential role in enhancing conventional therapies. TCM compounds could modulate a variety of cellular and molecular pathways, potentially inhibiting tumor proliferation, invasion, and metastasis. Besides, the practices of TCM alleviate the side effects of conventional treatments and enhance immune function, hence promoting the quality of life among lung cancer patients. In this regard, this review gives a contemporary account of the state of affairs on the part of TCM within the framework of the treatment of lung cancer with reference to its recent developments, and diverse roles.

## 1 Introduction

Lung cancer therapy has evolved to include a wide variety of approaches, including radiotherapy, chemotherapy, surgery, and other modalities [1]. A large percentage of lung cancer patients are now diagnosed at more advanced stages, potentially depriving them of the chance for surgical intervention [2]. Furthermore, the well-being of patients is significantly compromised due to the adverse effects of radiotherapy. These effects include cardiotoxicity, bone marrow suppression, skin problems, gastrointestinal disturbances, and severe fatigue [3]. Despite certain successes of chemotherapeutic agents in addressing lung cancer, numerous clinical observations underscore the existing limitations of current chemotherapy protocols. These limitations include poor prognosis, high recurrence and metastasis rates, and short survival [4]. Moreover, resistance to chemotherapy is often developed by lung cancer patients, which decreases the effectiveness of chemotherapy and shows the flaws of conventional treatments [4].

Of particular note is the widespread recognition of complementary and alternative medicine as a therapeutic approach to provide supportive and palliative care for cancer patients [5]. The United States is spending $33.9 million annually on complementary and alternative medicine [6]. Furthermore, cancer patients frequently turn to complementary and alternative medicine for support; notably, 25–50% of prostate cancer patients have engaged with at least one complementary and alternative medicine method [7]. Traditional Chinese medicine (TCM), a significant component of complementary and alternative medicine in Western societies, has also been utilized in cancer treatment [8]. The year 2011 witnessed a surge of interest in the potential applications of traditional Asian medicinal practices in cancer therapy, prompting investigations into their viability using modern scientific methodologies [9]. TCM has been employed in clinical settings to address symptoms associated with lung cancer rather than solely focusing on eliminating lung cancer cells, given the absence of the term “lung cancer” in ancient China [10]. In other words, TCM practitioners take a holistic approach in treating the patient, an approach that addresses all aspects of the patient’s health [11]. Significantly, the Yangzheng Xiaoji (YZXJ) capsule, a preparation based on TCM, showed anti-lung cancer effects [12]. A randomized, double-blind trial conducted in lung cancer patients found that those receiving YZXJ with conventional chemotherapy had higher rates of complete and partial remission of the disease than those receiving chemotherapy alone [12]. Clinical trials have shown that the Fuzheng Quxie Formula, a compound extract of TCM herbs, can significantly improve progression-free survival (PFS) and bolster immune function in patients with lung cancer [13]. It is composed of herbs with both immune-boosting and tumor-suppressing properties, acting through modulation of the tumor microenvironment (TME), reduction of systemic inflammation, and enhancement of anti-tumor immune responses [13]. Certain studies suggest that TCM extracts and multi-targeted prescriptions mitigate treatment resistance in lung cancer by impeding tumor growth, inducing tumor apoptosis, and restraining tumor proliferation [14–16]. As a result, this review aims to provide an overview of the current landscape, delve into recent advancements, methodologies, and roles of TCM in lung cancer treatment, and offer a potential avenue as an adjuvant therapeutic option.

## 2 TCM for cancer treatment

With its rich historical lineage and extensive expertise, TCM occupies a distinguished position in the realm of cancer management [17]. The initial phase of TCM treatment aims to optimize the state of healthy “Qi,” to enhance the survival of individuals grappling with tumors by impeding disease growth and alleviating associated symptoms. “Qi” (chee) is the vital life force or energy flowing through the body, according to TCM; it is the basic energy that sustains life and is believed to flow along specific pathways called meridians in the body [18]. A crucial disparity in efficacy becomes apparent when comparing TCM with modern medical approaches [19]. While TCM may not lead to substantial tumor shrinkage, patients often experience prolonged survival and significant improvements in subjective symptoms [20]. In contrast, contemporary therapies frequently result in observable tumor reduction, yet they are accompanied by swift recurrence, limited survival, and compromised quality of life [17].

Generally, TCM emphasizes the comprehensive management of a patient’s physiological processes at a macroscopic level [21]. The core objective of TCM therapy for tumors is to reinstate equilibrium within the internal milieu by adjusting internal factors. This approach seeks to augment the body’s ability to combat pathogens, thereby curbing the proliferation and dissemination of tumors [22]. Guided by pattern identification, traditional Chinese remedies capable of fortifying vital energy, boosting the body’s resilience, and addressing debilitation conditions become essential. This strategy aims to harmonize the interplay of yin, yang, Qi, and blood within the human body, alongside the physiological functioning of organs and meridians. In TCM, “yin” represents one of the two complementary forces associated with coolness, rest, and nourishment, in opposition to its opposite, “yang,” which represents warmth, activity, and energy; a balance between the two is necessary for health [23]. The ultimate goal is to elevate overall quality of life, enhance innate disease resistance, and modulate immune function. Consequently, integrating TCM into cancer treatment can potentially ameliorate overall health status, mitigate disease or pathogenic factors, impede tumor progression, alleviate symptoms, and prolong survival [24].

Over the past few decades, numerous clinical and laboratory investigations have been conducted to ascertain the effectiveness of TCM in cancer treatment. Various components derived from TCM have exhibited anti-cancer properties by effectively halting human cancer’s progression, proliferation, angiogenesis, and metastasis. Phytochemicals such as resveratrol, curcumin, and berberine are noted to have been extensively investigated in human studies for their therapeutic utility across a wide range of cancers [25]. TCM is mainly used as an adjuvant therapy coupled with the Western approach [26]. Innumerable studies have been published detailing patient outcomes due to the combination of TCM and chemotherapy. Oral supplementation of TCM has also been demonstrated to favorably affect the outcomes of chemotherapy in patients with non-small cell lung cancer (NSCLC), resulting in better quality of life, anemia, and neutropenia [27].

Curcumin, a bioactive metabolite isolated from the rhizomes of *Curcuma longa* L., is one of the major constituents in TCM [28]. One clinical trial in patients with pancreatic cancer showed that 8 g of oral curcumin given simultaneously with gemcitabine-based chemotherapy was a well-tolerated and potential treatment [29]. Notably, curcumin enhances the radiosensitivity of nasopharyngeal carcinoma cells by several mechanisms, including modulation of reactive oxygen species (ROS), regulation of Jab1/CSN5, and expression of noncoding RNA [30]. In addition, resveratrol and berberine have also been reported to enhance the radiosensitivity of nasopharyngeal carcinoma cells [31,32].

Recent years have seen the effective use of checkpoint immunotherapy using antibodies against cytotoxic T lymphocyte antigen-4 (CTLA-4), chimeric antigen receptor T cells, and programmed death-1 (PD-1) to modulate the immune system in its effort to eradicate cancer [33]. To this end, TCM has been suggested as an adjuvant to improve the results of immunomodulatory therapy for the treatment of cancers of various origins [34]. For example, curcumin treatment significantly increased the number of T-helper 1 cells in patients with colon cancer [35]. Co-administration of a curcumin-polyethylene glycol (PEG) compound with a vaccination effectively improved cytotoxic T-lymphocyte response and promoted interferon (IFN)-γ production [36]. In a mouse model of kidney tumors, modest dosages of resveratrol were observed to suppress tumor development through CD8^+^ T cell regulation [37]. As a result, numerous TCM components hold promise as potential partners for immunotherapy in comprehensive cancer treatment.

## 3 Major TCM compounds used in lung cancer therapy

Over the past many years, various herbs and their corresponding remedies/recipes have been utilized in China, South Asia, and East Asia to treat lung cancer [38]. *Astragalus membranaceus* has been observed to regulate programmed cell death, and apoptosis, in cancer cells [39]. This is attained through the upregulation of apoptosis-related proteins like caspase 3 and caspase 9 and high ratio of Bax to Bcl-2 proteins [40]. In a study, Zhou et al. [41] demonstrated that *A. membranaceus* inhibits proliferation in breast cancer cells by interfering with phosphatidylinositol-3-kinase (PI3K)/AKT/mammalian target of rapamycin (mTOR) pathway. Moreover, it was also demonstrated in the same study to induce apoptosis in NSCLC [42]. *Panax ginseng* is the dried rhizome of Araliaceous plant, traditionally reported to exert multitargeted pharmacological effects such as protection of cardiovascular system, anticancer, and antiaging properties [43]. Further investigation of human lung cancer cell line A549 revealed that ginsenoside Rg3 could induce apoptosis by increasing ROS production and upregulate the expression of some apoptosis-related proteins, including caspase 3/9 and BAX [44].

Recent research shows that Ganoderma triterpenoids can inhibit lung cancer cell growth. This inhibitory action is thought to be linked to cell cycle control and the enhancement of the Bax/Bcl ratio [45]. Triterpenoids have also demonstrated the capacity to downregulate matrix metalloproteinases (MMPs), thereby suppressing the dissemination of prostate cancer cells and curtailing cancer activity [46]. Extract from *Ganoderma lucidum* has proven effective in reducing the population of breast cancer stem cells (BCSCs) by suppressing the signal transducer activator of transcription 3 (STAT3) pathway, consequently inhibiting the invasive capacities of cancer cells [47]. Additionally, ethanol-extracted *G. lucidum* has been shown to enhance cellular autophagy mechanisms for protection while simultaneously suppressing the expression of epidermal growth factor receptor (EGFR) and the PI3K/AKT/mTOR signaling pathway in chronic myelogenous leukemia-affected cells [48].

Moreover, *G. lucidum* polysaccharides could also enhance anti-tumor activities of paclitaxel (PTX), one of the most commonly used chemotherapeutic drugs. In addition, it could inhibit cancer-related metabolic pathways in TME [49]. Apart from directly killing cancer cells, *G. lucidum* may also play a role in cancer immunotherapy. Notably, a composite material comprising *G. lucidum* polysaccharide and gold nanoparticles has demonstrated the ability to stimulate dendritic cells, enhance T cell proliferation, and effectively inhibit the proliferation and metastasis of lung cancer cells [50]. Research findings highlight that a significant concentration of *Angelica sinensis* can impede lung cancer cell metastasis. This mechanism involves not only the downregulation of MMP2, MMP-9, transforming growth factor beta-1 (TGFβ-1), and tissue inhibitor matrix metalloproteinase (TIMP)-1 but also upregulation of TIMP-2 expression [51]. *Panax notoginseng* has the potential to regulate macrophage M1 polarization and thus induce the apoptosis of lung cancer cells by the upregulated expression of apoptotic protein caspase 3/9 and thereby inhibit the solid tumor size [52]. Recent studies have focused on the strong inhibitory effect of ethanol-extracted tanshinone on gastric adenocarcinoma, lung adenocarcinoma, colorectal cancer (CRC), prostate cancer, and breast cancer [53–55].

## 4 Action mechanisms of TCM in lung cancer

**Figure 1:**
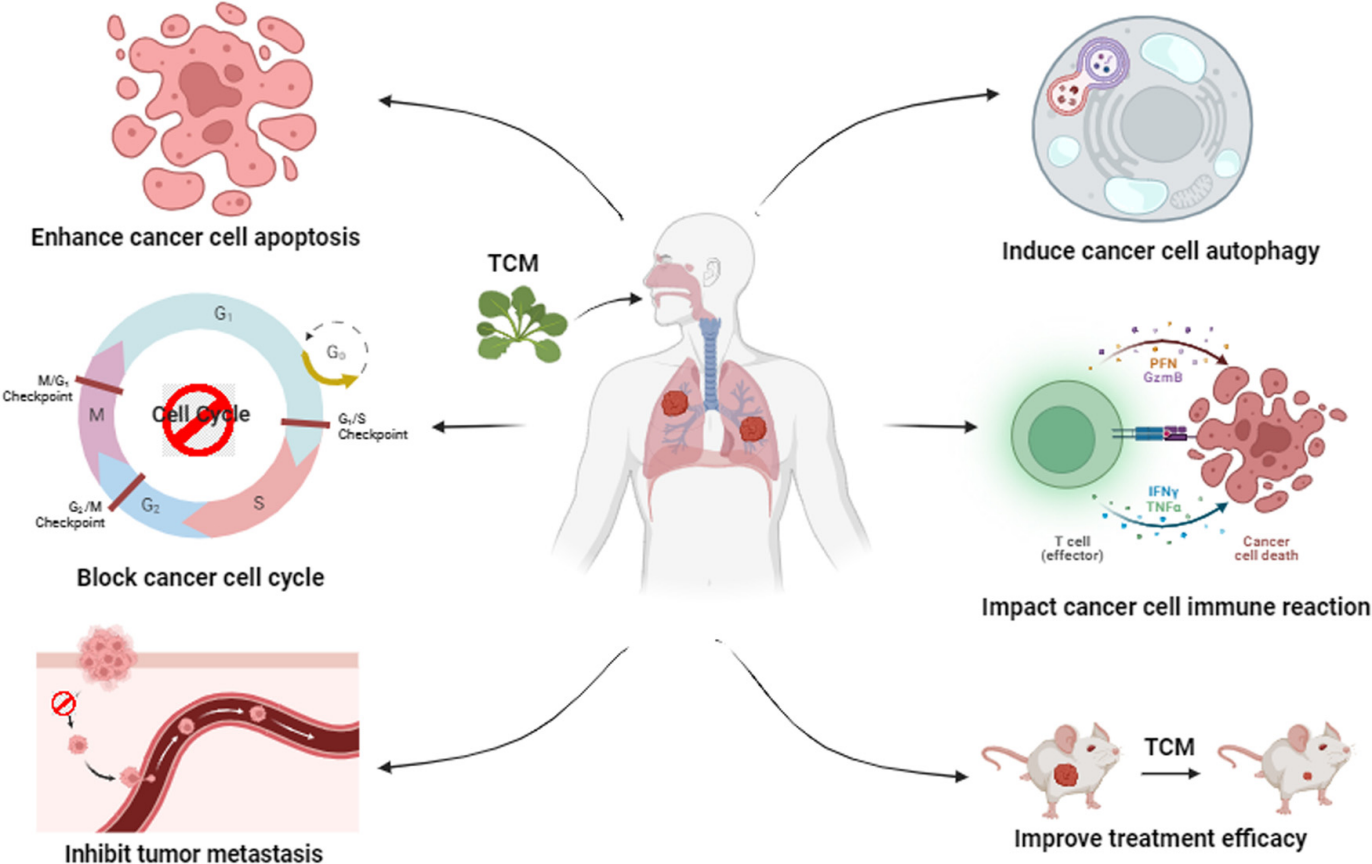
Role and action mechanisms of TCM in the treatment of lung cancer. TCM applies in the different fields of lung cancer, including apoptosis, metastasis, immunotherapy, autophagy, cell cycle regulation, and enhancement of treatment efficacy. TMC often contains bioactive compounds that show apoptotic activities toward lung cancer cells. Several herbs, including resveratrol, curcumin, and quercetin, were reported to induce apoptosis through regulating apoptosis-related signaling molecules, including caspases and Bcl-2 family proteins. TCM interventions do attempt to control metastatic spread of tumor cells by acting through the basis of factors influencing such metastatic processes. Some of the herbs commonly used in TMC, such as *A. membranaceus* and *S. baicalensis*, exhibit their anti-metastasis action through downregulation of MMPs, VEGF expression, and epithelial-mesenchymal transition (EMT) markers. The immune-modulating compounds isolated from the herbs of TCM, including *G. lucidum* (Reishi mushroom) and *P. ginseng*, increase the cytotoxicity of NK cells, promote the maturation of dendritic cells, and increase cytokine production. Some of the components of TCM have been found to modulate autophagy pathways that sensitize lung cancer cells to other treatments or inhibit their survival when in a stressful condition. Aberrant cell cycle progression is one of the hallmarks of cancer. TCM herbs contain phytochemicals, such as flavonoids and alkaloids, that may modulate the cell cycle by targeting cyclins, cyclin-dependent kinases, and checkpoint proteins, leading to cell cycle arrest and eventually cell death. TCM is often used as an adjuvant to conventional therapies, such as chemotherapy and radiotherapy, in order to enhance the effectiveness and reduce the side effects of these treatments. Some of the TCM compounds, such as those in *A. membranaceus* and *Camptotheca acuminata*, were reported to sensitize cancer cells to radiation and chemotherapeutic agents.

TCM formulations have shown great strides in the management of lung cancer (Figure 1). These formulae orchestrate the body’s physiological processes to holistically inhibit tumor growth and progressively exhibit their therapeutic virtues within clinical contexts. Numerous studies have unveiled that some TCM formulations possess the potential to influence signal pathways intricately linked with lung cancer. These prescriptions, in turn, successfully inhibit cancer cell growth by various mechanisms, including induction of apoptosis and cellular cycle arrest among others. Moreover, the use of TCM prescriptions in the treatment of tumor includes a variety of aspects. This includes inhibition of tumor cell invasion and metastasis, reversal of drug resistance, enhance the efficacy of chemotherapy, relieve the side effects of chemotherapy drugs, adjust the immune function, and inhibit the proliferation of cancer stem cells among other aspects. This section covers an overview of the various modalities by which TCM formulae are exploited to seek treatment of lung cancer (Tables 1 and 2).

**Table 1:** Role of TCM-derived components in lung cancer treatment, categorized by mechanism of action

TCM compound	Study setting	Cancer type	Mechanism	Target pathway	Conclusion	Ref.
Qiyusanlong decoction (QYSL)	*In vivo*	LLC	Inhibits tumor growth, decreases PD-1/PD-L1 levels	PD-1/PD-L1 pathway	Inhibits tumor growth by decreasing PD-1/PD-L1 expression, showing potential for combination therapy	[56]
Curcumin	*In vitro*	A549 cells	Inhibits proliferation, induces apoptosis	PI3K/AKT, lncRNA UCA1	Suppresses lung cancer proliferation	[57]
Curcumin	*In vitro* and *in vivo*	H157, H1299 (NSCLC)	Overcomes primary gefitinib resistance, induces autophagy-related cell death	EGFR, Sp1, ERK/MEK, AKT/S6K pathways	Sensitizes gefitinib-resistant NSCLC to EGFR-TKIs via autophagy-dependent mechanisms	[58]
Curcumin	*In vitro* and *in vivo*	NSCLC	Potentiates gefitinib effect, induces EGFR degradation, modulates p38 activation	EGFR phosphorylation, p38 MAPK	Enhances gefitinib’s anti-tumor activity, mitigates gastrointestinal side effects	[59]
Yu Ping Feng San (YPFS)	*In vitro* and *in vivo*	A549 and A549/DDP (cisplatin-resistant)	Increases cisplatin accumulation, modulates drug resistance	ABC transporters, GSTs, NF-κB, Bax/Bcl-2	Reverses cisplatin resistance by regulating transporter activities, promoting apoptosis	[60]
Yang-Yin-Jie-Du Decoction (YYJDD)	*In vitro* and *in vivo*	H1975 (gefitinib-resistant)	Reverses gefitinib resistance, increases apoptosis	PI3K/Akt signaling pathway	Overcomes gefitinib resistance by downregulating the PI3K/Akt pathway	[61]
Resveratrol	*In vitro* and *in vivo*	A549 cells	Modulates long noncoding RNAs (lncRNAs), induces cell cycle arrest	AK001796, G0/G1 phase arrest	Decreases cell viability, slows tumorigenesis	[62]
*Scutellaria baicalensis*	*In vitro*	A549 and H1299	Inhibits proliferation, metastasis, and inflammation	Bcl-2/Bax, MMP-2, MMP-9, NF-κB	Counteracts nicotine-induced tumor progression	[63]
Wogonin	*In vitro*	A549	Induces apoptosis, inhibits oncogenic proteins	c-Myc/SKP2/Fbw7α, HDAC1/HDAC2	Effective in drug-resistant cancers	[64]
Silybin	*In vitro* and *in vivo*	A549	Inhibits SIRT1	SIRT1 signaling	Suppresses lung adenocarcinoma growth	[65]
Silibinin	*In vitro* and *in vivo*	NSCLC	Acts as an HDAC inhibitor	Epigenetic regulation (HDACi)	Promising combination with HDAC inhibitors for NSCLC	[66]
Celastrol	*In vitro* and *in vivo*	NSCLC	Inhibits EGFR pathway	EGFR-TKI resistance	Suppresses invasion in T790M-mutant lung cancer	[67]
JP-1	*In vitro*	A549	Induces apoptosis	p53/miR-34a pathway	Suppresses proliferation, resistance, and metastasis	[68]
Yangyinwenyang (YYWY)	*In vitro*	NSCLC	Enhances dendritic cell (DC) maturation	Immune modulation	Stimulates T-cell response against NSCLC	[69]
Total flavonoids from *Adinandra nitida* Merr. ex Li leaves (TFAN)	*In vitro* and *in vivo*	NSCLC	Induces apoptosis, disrupts NADPH homeostasis, increases ROS	ROS-mediated p53 activation	Suppresses NSCLC growth via apoptosis induction	[70]
QDN	*In vitro* and *in vivo*	NSCLC	Induces apoptosis via mitochondrial fission	p53/DRP1-mediated mitochondrial fission	Inhibits proliferation, increases ROS, promotes apoptosis via p53 activation	[71]
JFA Decoction	*In vitro* and *in vivo*	NSCLC	Inhibits proliferation, invasion, and metastasis	PI3K/Akt, Lumican/p120ctn, RhoGTPase	Suppresses tumor growth by blocking PI3K/Akt and modulating Rho family members	[72]
JFK	*In vitro* and *in vivo*	NSCLC	Inhibits metastasis, modulates immune response	T cell receptor (TCR) regulation, CD8^+^ T cell and NK cell activation	Suppresses tumor progression by enhancing immune infiltration, reversing TCR changes, and reducing MDSC-mediated immune suppression	[73]
Huaier	*In vitro* and *in vivo*	NSCLC	Suppresses cisplatin resistance, cancer stemness	JNK/JUN/IL-8 signaling pathway	Suppresses cisplatin resistance by inhibiting JNK phosphorylation and IL-8 expression	[74]
β-Sitosterol	*In vitro*	A549/anlotinib-resistant cells	Enhances sensitivity to anlotinib, promotes apoptosis	miR-181a-3p/SHQ1 signaling pathway, UPR activation	Inhibits miR-181a-3p, promotes apoptosis, and enhances sensitivity to anlotinib by activating SHQ1/UPR signaling	[75]

**Table 2:** Summary of clinical trials of TCM in lung cancer

TCM compound	Study setting	Cancer type	Mechanism	Target pathway	Efficacy results	Toxicity evaluation	Pharmacokinetics (PK)	Conclusion	Ref.
Haishengsu	Randomized, double-blind, placebo-controlled trial	NSCLC	Improves chemotherapy efficacy, reduces nausea/vomiting	Chemotherapy adjuvant	Enhances chemotherapy efficacy and reduces side effects	Mild-to-moderate nausea and vomiting in patients	Not specified	Effective supplementary treatment with chemotherapy for NSCLC	[76]
Feitai	Randomized controlled trial	NSCLC	Improves quality of life	Supportive care	Enhances quality of life in mild-to-late stage NSCLC	No significant adverse effects observed	Not specified	Improves the quality of life in advanced NSCLC stages	[77]
Unnamed TCM regimen	Randomized, controlled clinical trial	NSCLC	Maintains disease stability	Maintenance therapy	Comparable efficacy to single-agent chemotherapy	No notable toxicity	Not specified	Comparable efficacy to chemotherapy, beneficial for disease stability	[78]
Curcuma zedoaria, Shelled Walnut, Folium Eriobotryae, etc.	Prospective cohort study	Extensive-stage small cell lung cancer (SCLC)	Prolongs PFS and post-progression survival (PPS)	Multiple pathways	Significant prolongation of PFS and PPS	No severe toxicity reported	Not specified	Enhances survival and stabilizes patient conditions in SCLC	[79]
Qi and Yin deficiency treatment	Double-blind controlled, multi-center, prospective study	NSCLC	Improves PFS, enhances overall survival	Qi and Yin deficiency treatment	Potential improvement in PFS, overall survival, and quality of life	To be assessed (toxicity, side effects, safety)	Not specified	Investigates the combination of chemotherapy with TCM for elderly patients with advanced NSCLC, focusing on PFS and overall survival	[80]
Chinese medicine + adjuvant chemotherapy (NP/NC)	Randomized controlled trial	Early-stage NSCLC	Reduces side-effects, enhances quality of life	Chemotherapy adjuvant	Partial relief of symptoms, reduced side effects	Lower incidence of adverse events in the intervention group, less severe hematological toxicity	Not specified	TCM combined with chemotherapy reduces adverse events and improves quality of life	[81]
Chinese Herbal Medicine Formulas (CHMF) + adjuvant chemotherapy	Randomized, double-blind, placebo-controlled trial	Lung adenocarcinoma (LAC)	Reduces chemotherapy-related toxicity, enhances disease-free survival (DFS)	Chemotherapy adjuvant	Improved DFS in early-stage patients, no significant OS difference	Decreased incidence of dry mouth, diarrhea, fatigue, and thrombocytopenia in the CHMF group	Not specified	CHMF combined with chemotherapy improves DFS and reduces chemotherapy-related side effects in LAC patients after surgery	[82]
TCM + chemotherapy	Retrospective cohort study	Stage II-IIIA NSCLC	Reduces recurrence, metastasis, enhances survival	Chemotherapy adjuvant	Prolonged DFS in TCM group, significantly better in high-risk patients	Not specified	Not specified	TCM combined with chemotherapy reduces recurrence, metastasis, and enhances DFS in stage II–IIIA NSCLC patients after surgery	[83]
TCM + EGFR-TKI	Prospective clinical trial	EGFR-mutant NSCLC	Enhances PFS, no additional adverse effects	EGFR mutation treatment	Significant increase in mPFS for both exon 19 and exon 21 mutations	No additional adverse effects compared to EGFR-TKI alone	Not specified	TCM combined with EGFR-TKI significantly prolongs PFS in EGFR-mutant NSCLC patients without additional adverse effects	[84]
TCM	Randomized controlled trial	Advanced NSCLC	Prolongs time to progression (TTP), enhances quality of life (QOL), improves 1-year survival rate	Maintenance therapy	No significant difference in TTP, improved QOL, higher 1-year survival rate (78.1% vs 53.1%)	Not specified	Not specified	TCM maintenance treatment improved QOL and 1-year survival in advanced NSCLC patients without progression after first-line chemotherapy	[85]
TCM treatment on serum sCTLA-4	Prospective randomized controlled trial	Advanced NSCLC	Regulates serum concentration of sCTLA-4 to influence PFS	Immune modulation (sCTLA-4 regulation)	Significant decrease in sCTLA-4 levels, improved prognosis	No significant adverse effects compared to chemotherapy	Not specified	TCM regulates sCTLA-4 levels and improves TTP in advanced NSCLC patients	[86]

### 4.1 TCM in lung cancer apoptosis

TCM demonstrates its capability in inducing apoptosis of lung cancer cells through the activation of various proteins and apoptotic signal pathways. The mechanistic studies in TCM are mainly focused on TCM monomers. Cantharidin (CTD), an odorless lipid extracted from mylabris species, was regarded as a potent TCM monomer, which exhibited profound cytotoxicity on various cancerous cells [87]. Incubation of CTD has been shown to reduce viable cell percentage and induce morphological changes in human H460 lung cancer cells. Genetic over-expression of caspase-3 and caspase-8 due to CTD is further correlated with an increase in the production of ROS along with calcium ions (Ca^2+^). It also upregulates the Bax, cytochrome c, and apoptosis-inducing factor levels, with a corresponding decrease in Bcl-XL expression [87]. Moreover, increased CTD levels cause endoplasmic reticulum (ER) stress, along with the inhibition of calpain 1, a protein that plays a significant role in regulating apoptotic pathways and protein expression. These results emphasize the fact that CTD can trigger apoptosis in lung cancer cells via the mitochondria [87]. All these findings suggest that CTD is an effective apoptosis-inducing agent for the treatment of lung cancer, primarily through mitochondrial and ER stress-mediated pathways.

*Clematis flammula* L. (CFI), an herbaceous plant native to North Africa and Southern Europe, is an important medicinal material in TCM [88]. CFI is used as a treatment for rheumatism in the Chinese populace. Recent understanding reveals that boehmenan, a constituent of CFI, induces apoptosis in lung cancer cells by regulating epidermal growth factor (EGF) dependent pathways [89]. In the context of breast cancer cells, Clematis hederagenin saponin (CHS), derived from CFI has been shown to induce apoptosis via the mitochondrial route [90]. In a separate experimental study, HCT116 colon cancer cells underwent combined treatment involving CFI and tumor necrosis factor (TNF)-related apoptosis-inducing ligand (TRAIL). Notably, the combination showed a synergistic effect in comparison with either CFI or TRAIL alone, as indicated by the downregulation of cell survival proteins implicated in the apoptotic process. These findings underscore CFI’s ability to increase the sensitivity of TRAIL-resistant cells to apoptosis. This sensitization is mediated through the activation of SP1, mitogen-activated protein kinase (MAPK), and CCAAT/enhancer-binding protein homologous protein (CHOP), which are established regulators of DR5 expression [91]. Taken together, these observations suggest that CFI and its bioactive constituents induce potent pro-apoptotic activity in cancer cells from a variety of histological origins via both mitochondrial and receptor-mediated pathways of apoptosis.

Bufalin is an oriental cardiotonic steroid originally isolated from toad venom, which has shown potent activity in lung cancer NCI-H460 cell, inducing a loss of mitochondrial membrane potential and increased ROS generation [92]. Treatment with Bufalin upregulated proapoptotic proteins and downregulated anti-apoptotic proteins. Hence, it was proven that Bufalin can induce apoptosis in lung cancer cell lines *in vitro* and reduce the size of tumors owing to its anticancer characteristics [92]. Cryptotanshinone, a bioactive compound extracted from *Salvia miltiorrhiza* Bunge, has been found to induce apoptosis in A549 lung cancer cells at a concentration of 20 μmmol/L. This effect was coupled with upregulated Bax and P53 expression and downregulated Bcl-2 levels [93]. Similar results were achieved from *in vitro*, which illustrated the ability of cryptotanshinone to inhibit tumor growth and induce apoptosis. This confirms the therapeutic value of cryptotanshinone in TCM concerning the prevention of lung cancer and induction of apoptosis, as supported by *in vitro* experiments and animal models [93].

Gmiscorrhizae, one of the active constituents of *P. ginseng* C.A. Meyer, contains numerous phytoconstituents, such as CK, CMc, CMc1, CO, CY, Rb1, Rb2, Rc, Rd, F2, Rg3, and Rh2, primarily of protopanaxadiol (PPD)-type ginsenosides [94]. Unexpectedly, CK, an active ginsenoside metabolite in the blood and urine, promotes apoptosis induced by gamma ray in human lung cancer cells via generation of ROS and depolarization of mitochondrial membrane [95]. Magnolol, a compound from Magnolia officinalis Rehder, was reportedly found to exhibit multiple pharmacological effects, including anti-depressive, antioxidant, anti-inflammatory, and anti-tumor activities [96]. Interestingly, magnolol or a polyphenol mixture (PM) induced increased expression of pro-apoptotic protein Bax and downregulated anti-apoptotic protein Bcl-2 simultaneously. The treatment with magnolol and PM also upregulated caspase-3, poly(ADP-ribose) polymerase (PARP) cleavage protein, and caspase-3 cleavage expression in A549 and H1299 cells [97]. In general, ginsenoside CK and magnolol exert potent pro-apoptotic effects on lung cancer through the induction of ROS production, mitochondrial function disruption, and modulation of apoptotic protein expression, showing great potential as promising TCM-derived anti-cancer therapeutics.

Cinnamaldehyde, the major organic compound of Cinnamomum cassia’s essential oil, has been shown to induce apoptosis in lung cancer cells through various pathways [98]. It induces programmed cell death in lung cancer cells and inhibits EMT through the Wnt/β-catenin signaling pathway [99]. Puerarin, an isoflavone isolated from Kudzu roots, showed inhibitory effects on NSCLC cell growth. Results showed that puerarin-induced apoptosis in NSCLC through the mitochondrial-mediated apoptosis pathway thus holds promise for the treatment of lung cancers [100]. Fuzheng Kang-Ai (FZKA) supported apoptosis in lung cancer cells by enhancing the activity of caspase-3, caspase-9, and PARP. FZKA also disrupted the levels of Bcl-2 family proteins and notably reduced the expression of STAT3, thereby promoting apoptosis via the Bcl-2/caspase-3/STAT3 pathways [101]. Xiaoji decoction (XJD), a blend of Chinese herbs, demonstrated potential in restraining lung cancer cell proliferation by utilizing AMP-activated protein kinase (AMPK) α to suppress DNA methyltransferase 1 and transcription factor Sp. It triggered apoptosis in A549 cells by increasing caspase-9 and BAD expression through the protein kinase B (Akt) pathway, with effects dependent on XJD dosage and treatment duration [102,103]. One recent study by Lan et al. [70] was conducted to investigate the effects of total flavonoids from *Adinandra nitida* (TFAN) on NSCLC apoptosis and its molecular mechanisms. They demonstrated that TFAN induces apoptosis in NSCLC cells by disrupting nicotinamide adenine dinucleotide phosphate (NADPH) homeostasis, thus elevating ROS production and further activating p53 and eventually causing apoptotic cell death and selectively inhibiting NSCLC growth [70]. Besides, QiDongNing (QDN) was indicated to be a powerful apoptosis inducer in NSCLC by targeting mitochondrial dynamics via the p53/DRP1 signaling pathway. Ding et al. [71] showed that QDN could markedly induce the apoptosis of lung cancer cells, which is closely related to mitochondrial dynamics. QDN exerted its actions on the structure, function, and metabolism of mitochondria by inducing an increase in mitochondrial fission via the p53/DRP1 pathway, hence ultimately resulting in the apoptosis of lung cancer cells [71]. In brief, cinnamaldehyde, puerarin, FZKA, TFAN, QDN, and XJD could notably suppress the growth of lung cancer cells through modulation of key signaling pathways, including Wnt/β-catenin, mitochondrial apoptosis, Bcl-2/STAT3, and Akt, thus further consolidating their potential as TCM-based therapeutic strategies.

### 4.2 TCM in lung cancer metastasis

Metastasis is the spread of cancerous cells from the original site to other distant parts of the body, which always occurs via the lymphatic or circulatory systems and results in the formation of secondary tumors [104,105]. The dissemination of cancer is generally believed to be a hallmark that endows malignancies with their deadly characteristics. Lung cancer metastasis refers to the process of spreading lung cancer cells from the original lung tumor to adjacent tissues, distant organs, or other anatomical locations in the body, often aided by the circulatory or lymphatic systems [106]. Metastasis has a very profound effect on patients’ morbidity and mortality rates with lung cancer. TCM acts on two fronts simultaneously: it inhibits the growth of malignant tumors and prevents the proliferation of cancer cells. EMT is one of the main physiological processes implicated in the invasion and metastasis of cancer cells, contributing largely to the dissemination of cancer cells.

TCM has found extensive application in the clinical care of cancer patients across all disease stages. Its deployment has yielded noteworthy improvements in efficacy and quality of life, alongside symptoms and prognoses amelioration. Furthermore, TCM has demonstrated the capacity to impede cancer development and metastasis [2]. Previous studies indicate that oxymatrine may suppress liver metastasis CRC through the inhibition of the GLUT1/pyruvate kinase muscle isozyme M2 (PKM2)-mediated aerobic glycolytic pathway [107,108]. Betulinic acid is a pentacyclic triterpene from *Betula mandshurica* Nakai, with reported strong anti-tumor effects. In a separate investigation, it was demonstrated that betulinic acid inhibits pulmonary metastasis in breast cancer by upregulating glucose-regulated protein 78 (GRP78) and downregulating lactate dehydrogenase A (LDHA) and pyruvate dehydrogenase kinase 1 to inhibit lactate production [109]. The primary bioactive agent underpinning *P. ginseng*’s anti-tumor prowess is Ginsenoside Rh2. Earlier studies spotlight Ginsenoside Rh2’s inhibitory effects on glycolysis modulated by GLUT1, PKM2, and LDHA, as well as on EMT processes in NSCLC, orchestrated through STAT3/c-MYC pathway control [110]. Icaritin, a bioactive compound sourced from desiccated epimedium stems and leaves, dampens glycolytic processes mediated by GLUT1 in hepatocellular carcinoma (HCC) cells through the impediment of Janus kinase 2 (JAK2)/STAT3 pathway activation. As a result, proliferation and migration of HCC cells are suppressed [111]. Moreover, Shikonin is a bioactive compound with anti-tumor activity isolated from the Chinese medicinal plant *Lithospermum erythrorhizon*, which is reported to exhibit inhibition effects on glucose uptake, lactate formation, and ATP synthesis in both lung cancer and melanoma cells. This is attributed to the downregulation of the expression of PKM2, leading to induce apoptosis and diminish proliferation and migration of cancer cells [112]. According to a study, the administration of modified Kejinyan decoction promoted M1-type macrophage polarization by downregulating TNF-α and IFN-γ. The inhibition of GLUT1-mediated glycolytic process in M1-type macrophages was also found to lead to suppressing lung cancer cell migration [113]. Generally, TCM-based compounds impose anti-cancer effects on glycolytic metabolism through modulating key metabolic pathways and immune responses.

Studies have demonstrated that the early stages crucial for metastasis are characterized by the clonal burst expansion of hematopoietic stem and progenitor cells within the bone marrow and subsequent differentiation of these stem and progenitor cells into myeloid-derived suppressor cells (MDSCs), in the early metastatic niches [114]. These MDSCs – bone marrow cell (BMC)-derived – comprise a heterogeneous population of myeloid progenitor cells, and a small proportion of these cells is found to support metastasis of cancers. MDSCs can be classified into two main subtypes based on the expression of Ly6G and Ly6C markers: granulocytic (CD11b^+^ Ly6C^+^ Ly6G^+^) and monocytic MDSCs (CD11b^+^ Ly6C^+^ Ly6G^−^) (known as G-MDSCs and M-MDSCs, respectively) [115,116]. These subgroups are believed to use distinct mechanisms to suppress T-cell responses, utilizing nitric oxide and ROS to induce immunosuppression [117].

In cancer contexts, MDSCs’ functions are influenced by factors produced by both the tumor and normal host cells. These factors create a favorable environment for tumor cell survival within a premetastatic niche, essential for tumor cells’ subsequent survival and growth in distant organs [118]. Furthermore, MDSCs can directly stimulate cancer cell growth, progression, and metastasis by overactivation of the mTOR pathway. Wei et al. [119] observed that Shuangshen granules (SSG), a blend of American ginseng, notoginseng, and cordyceps, significantly reduced MDSC levels in the bone marrow, blood, and lungs of tumor-bearing mice. Co-culture experiments showed that SSG directly impacted BMC differentiation, inhibiting CD11b^+^ Ly6C^+^ Ly6G^+^ cell formation *in vitro*. Intriguingly, similar effects were seen *in vivo* for CD11b^+^ Ly6C^+^ Ly6G^+^ cells but not for CD11b^+^ Ly6C^+^ Ly6G^−^ cells [119]. Given the role of the mTOR pathway in regulating myelopoiesis in the bone marrow and controlling abnormal differentiation into CD11b^+^ Ly6G^+^ cells, it is plausible that SSG affects the mTOR signaling pathway. Wei et al. [119] identified protein markers linked to the mTOR pathway in the bone marrow and lungs, noting significant reductions in mTOR/S6K1/Myc expression levels with SSG treatment. Additionally, SSG effectively impeded lung metastasis in a mouse model of lung cancer (Lewis lung cancer [LLC] xenograft), aligning with the fact that immature myeloid cells, including CD11b^+^ Ly6C^+^ Ly6G^−^ monocytes and CD11b^+^ Ly6C^+^ Ly6G^+^ granulocytes, significantly contribute to pre-metastatic niche formation in the lungs. These cells mediate immunosuppression, aid tumor cell colonization, and enhance pulmonary metastasis [114].

SSG components’ anti-tumor and anti-metastatic properties have been substantiated through *in vitro* investigations. Ginsenosides such as Rg3, Rh2, Rg5, ginsenoside K, and their derivatives have demonstrated the capacity to modulate signaling pathways related to tumor growth and spread. Notably, their inhibitory effects on tumor development may be due to their ability to induce apoptosis in tumor cells, promote tumor cell differentiation, selectively target cancer stem cells, and influence the TME by inhibiting proteins and pathways associated with tumor angiogenesis [120]. Polysaccharides from ginseng leaves have been reported to possess antimetastatic effects by increasing the activation of macrophages and natural killer (NK) cells [121]. *P. notoginseng* and its active compounds have been shown to inhibit tumor growth and metastasis in various tumors, which are evident in both *in vitro* and *in vivo* experiments [122,123]. Several SSG constituents, including R1, Rb1, and cordycepin, can modulate mTOR-related signaling pathways [124]. In Wei et al. [119] preliminary investigations, they also observed that SSG extracts significantly suppressed tumor cell growth. In addition, the results of both the *in vitro* and the *in vivo* tests showed that SSG can efficiently hinder the differentiation of BMCs into MDSCs and prevent the incidence of lung metastasis. The inhibition was obtained through the restraint of the mTOR pathway in bone marrow microenvironment [119]. These findings suggest that SSG could potentially impede the dissemination of cancer cells to specific organs by restraining the generation of MDSCs. The outcomes reported by Wei et al. [119] imply that SSG might emerge as a promising pharmaceutical option for preventing organ-specific metastasis in patients with early-stage NSCLC. SSF components have demonstrated strong anti-tumor and anti-metastatic activities by modulating apoptosis, tumor differentiation, activation of immune cells, and mTOR-related signaling. Their inhibition of MDSC differentiation identifies these compounds as promising approaches in the early prevention of organ-specific metastasis in NSCLC and warrants further molecular studies. Finally, Jinfu’an Decoction (JFK) exhibited good therapeutic effects in patients with NSCLC through modulation of the key molecular pathways driving cancer progression. Recent studies with network pharmacology and experimental verification have elucidated some mechanisms by which JFK may inhibit metastasis in lung cancer. Peng et al. [72] found that JFK modulates PI3K/Akt signaling, Lumican/p120ctn expression, and Rho GTPase activity.

### 4.3 TCM in lung cancer immunotherapy

The domain of immunotherapy has seen significant strides, precipitating a paradigm shift in the treatment of various types of cancers. TCM is increasingly being recognized as an important medicinal source in designing new strategies against cancers, including immunotherapy [125]. Considerable evidence exists to suggest that the modulation of tumor-associated macrophages (TAMs) by modulating their pro-tumoral activity is at the behest of advancing metastatic events via the synthesis of various molecules such as cytokines [126]. Previous studies have furnished proof indicating that dihydroisotanshinone I (DT), an active bio-molecule found in danshen, effectively impedes the tumor-promoting potency of macrophages in prostate cancer by suppressing the CCL2/STAT3 pathway [127]. It is also noteworthy that DT might exert analogous effects across various cancer types. Hence, Wu et al. [10] explored the influence of dynamic therapy on the interaction between macrophages and lung cancer cells. They implanted human A549 cells into nude mice by injecting them to develop a xenograft animal model. Before establishing the *in vivo* model, their objective was to authenticate the impacts of DT on the interchange between human lung cancer cells and murine RAW 264 cells. The cellular traits observed in the interaction between human lung cancer cells and human THP-1 cells resemble those encountered in intercellular communication. Hence, both human THP-1 cells and RAW 264 cells were employed to study the effects of DT on macrophages, totaling seven cell types. Furthermore, Wu et al. [10] executed a study to delve into DT’s direct consequences on lung cancer cells’ migratory potential. Moreover, they found the repercussions of cytokines discharged from a conditioned medium derived from THP-1 or RAW264.7 cells that underwent treatment with dimethyl sulfoxide or DT on the migratory capacity of lung cancer cells. Preceding research has largely emphasized the importance of direct physical contact in facilitating cellular associations. For instance, direct co-culture of macrophages with cancer cells has demonstrated dramatic enhancement of STAT3 activation in various types of cancers [128–130]. In order to more closely mimic the physiological interaction between macrophages and tumor cells, Wu et al. [10] used a direct mixed cell-cell co-culture system to investigate the motility of lung cancer cells and further elucidate the underlying signaling cascade. A previous study highlighted that the reciprocal communication between TAMs and lung cancer cells through the CCL2/CCR2 signaling pathway is a significant mechanism underpinning TAM-driven facilitation of lung cancer proliferation and dissemination [131]. Furthermore, tanshinone IIA exhibits cardioprotective attributes via restraining CCL2 and TGF-β1 release from cardiac fibroblasts. However, the impact of DT on cytokine secretion from lung cancer cells and macrophages remains incompletely elucidated [132]. Hence, Wu et al. [10] harnessed a human cytokine array to ascertain cytokines manifesting varied expression in the A549 cells treated with DT media. Their findings showcased decreased CCL2 and CXCL1 expression in A549 cells exposed to DT. Moreover, DT was found to curtail CCL2 production from macrophages. Furthermore, the migratory potential of A549 cells displayed partial restoration following the introduction of 5 pg/mL of CCL2 into the conditioned media. Prior research has highlighted the significance of CXCL1 as a cytokine in lung cancer progression [133,134]. As per Wu et al. [8] findings, the evidence underscores CCL2’s pivotal role as a cytokine in modulating the migratory capability of lung cancer cells subjected to DT. Notably, this investigation revealed heightened expression of IL-8. A former study demonstrated a substantial surge in IL-8 and CCL2 levels within A549 cells. Furthermore, repressing CCL2 expression induced a marked reduction in A549 cell proliferation, though IL-8 levels remained unaltered [135]. In summary, these findings show DT as a potential candidate for lung cancer therapy by disrupting macrophage-cancer cell crosstalk and suppressing key cytokines like CCL2. The detailed mechanisms underlying DT effects on cytokine regulation are not clear and require further investigation.

In a study by Luo et al. [136], they validated that Yu-Ping-Feng (YPF), a traditional Chinese herbal decoction, significantly curtails the progression of LLC tumors and extends the lifespan of mice carrying these tumors. YPF also appears to induce infiltration of NK cells into the tumor, elevate the NK cell population in the spleen, and enhance NK cell-mediated cytotoxicity. The suppression of tumor growth achieved by YPF can be notably reversed by reducing NK cell levels. Subsequent investigations have revealed that YPF substantially diminishes the production of TGF-β, indoleamine 2,3-dioxygenase (IDO), and IL-10, thereby directly or indirectly modulating NK cell activity. This suggests that YPF might attenuate NSCLC through NK cell regulation. In the study by Luo et al. [136], YPF significantly stimulates NK cell infiltration into tumors, increases the NK cell population in the spleen, and enhances NK cell-mediated tumor-killing capabilities. On the other hand, reducing the count of NK cells lessens the suppressive impact of YPF on tumor growth, presenting strong proof that YPF has the potential to boost NK cell performance in mice with tumors. Thus, YPF might be able to act as a viable immune-modulating remedy for cancer immunotherapy. Inside the TME, diverse agents discharged by cancer cells, cancer-associated stroma, or immune cells can hinder NK cell operation [137]. Cancer cell-produced TGF-β, regulatory T cells, and tumor-associated fibroblasts can inhibit NK cell-driven antitumor reactions [138,139]. Additionally, IDO, generated as a response to IFN-γ by endothelial cells, mesenchymal stromal cells, fibroblasts, and various myeloid-derived cells, including dendritic cells and macrophages, can diminish NK cell function [140,141]. While IL-10, an immunosuppressive cytokine, does not directly limit NK cell activities, it can indirectly hinder NK cells by suppressing the release of IL-12, IL-15, and IL-18 through its supporting cells [142]. In the study by Luo et al. [136], they observed that YPF downregulates TGF-β, IDO, and IL-10 in tumor tissues, which could partially explain the boosted NK cell activity attributed to YPF. Recently, Fei-Liu-Ping, another Chinese herbal treatment, demonstrated its ability to suppress LLC, not only the primary tumor but also lung metastases, indicating that YPF might also have inhibitory effects on lung metastasis [143]. These results pinpointed YPF as an immune-modulating therapy with the potential to treat lung cancer. The anti-LCC effects of YPF were mediated through enhanced NK cell activity, increasing infiltration into tumors and cytotoxicity. Besides, YPF downregulates immunosuppressive factors like TGF-β, IDO, and IL-10, further enhancing NK cell function. All of these results mean that YPF would inhibit not only the growth of primary tumors but also metastasis, showing promise as a candidate for NSCLC immunotherapy. Also, in another study, Jinfukang exhibited potent anti-metastatic activity in lung cancer by modulating T-cell immunity, reversing altered TCR, and increasing CD8^+^ T and NK cell responses. Luo et al. [73] showed that JFK increases the infiltration of cytotoxic immune cells, namely CD8^+^ T lymphocytes and NK cells, in lung tumors. Moreover, JFK influenced the composition of immune cells in peripheral blood by increasing the percentages of CD4^+^ T, CD8^+^ T, and invariant NKT cells while modulating MDSCs and macrophage populations in a way that supports anti-tumor responses [73]. At the molecular level, the progression of lung cancer is associated with specific alterations in the TCR repertoire, including the downregulation of TRBV16, TRBV17, and TRBV1 and the upregulation of TRBV12-2. Changes were reversed in JFK treatment, restoring a more effective TCR profile for strong T-cell-mediated tumor surveillance [73]. These data show that, apart from enhancing the level of activity of immune cells, JFK might also reprogram the immune system to overcome tumor-driven immune suppression.

### 4.4 TCM and regulation of lung tumor-associated inflammation

Tumor-associated inflammation involves complex interactions between epithelial and stromal cells that, under the right conditions, can induce epigenetic modifications that promote neoplastic evolution and, occasionally, may serve as an initiation event for carcinoma [144]. This chronic inflammation helps to release additional growth factors capable of promoting proliferation in nascent tumors. The continuous secretion of various cytokines, chemokines, and growth factors within the TME sustains most of the cancer cells’ life cycle aspects, including proliferation, evolution, survival, tumor vascularization, and dysregulation of the immune system. All these combined create an enabling environment that furthers tumor advancement, invasion, and metastasis, developing resistance to therapy [145]. Bioactive TCM constituents may also modulate cytokine expression, regulate inflammatory inflammasomes, and nuclear factors, which further exert antitumor-regulating effects on inflammation.

In a study by Ma et al. [146], they revealed that the inhibitory effects of salidroside (SAL) on the proliferation and migration of A549 cells treated with lipopolysaccharide (LPS) are mediated by its capacity to activate AMPK, subsequently reducing NLRP3 inflammasome activation with the initiation and progression of tumors. Recent evidence underlines that patients with NSCLC bearing concurrent bacterial infections have exaggerated inflammatory responses [147,148]. Notably, NSCLC patients with bacterial infections have shown heightened activation of the TLR4/IL-33 axis, which promotes tumor growth [149]. In metastatic NSCLC patients, stimulation of blood cells with LPS increased the levels of IL-6 and IL-18, which is associated with clinical outcomes [150]. These results suggest that intervention in inflammatory signaling in NSCLC, specifically in patients with bacterial infections, may have a therapeutic potential for slowing tumor development.

Increasing evidence points toward SAL’s anti-inflammatory effects stemming from its potential to inhibit the NLRP3 inflammasome. In mice with carbon tetrachloride-induced acute liver injury, SAL significantly reduced NLRP3 inflammasome activation and the severity of liver injury [151]. Similarly, SAL administration also improved lung injury induced by mechanical ventilation in mice through inhibition of the NLRP3 in a Sirt1-dependent way [152]. In ulcerative colitis models induced by dextran sulfate sodium, SAL’s defensive effects were partly linked to its capability to hinder the NLRP3 inflammasome. Likewise, SAL treatment improved lung injury caused by mechanical ventilation in mice by inhibiting the NLRP3 inflammasome in a Sirt1-dependent manner [152]. In ulcerative colitis models induced by dextran sulfate sodium, SAL’s defensive effects were partly linked to its capability to hinder the NLRP3 inflammasome [153]. Moreover, SAL has been shown to control the NLRP3 inflammasome through the TXNIP–NLRP3 pathway, offering safeguarding against elevated glucose exposure that triggers excessive extracellular matrix accumulation in glomerular mesangial cells or insulin resistance in hepatocytes [154,155]. Taken together, these results suggest that the wide-ranging anti-inflammatory effects of SAL in a series of models of inflammation are largely explained by its inhibition of the NLRP3 inflammasome through multiple mechanisms.

Recently, a study conducted by Wang et al. [156] unveiled that SAL plays a role in controlling the production of ROS and the p38 MAPK signaling pathway. This leads to a reduction in the growth, cell cycle progression, and metastasis of A549 cells while promoting apoptosis. Furthermore, SAL has exhibited the capacity to diminish the survival, migration, and invasion of A549 cells by suppressing the Akt and MEK/ERK signaling pathways and enhancing the expression of microRNA-195 [157]. Another study by Ma et al. [146] also indicated that SAL efficiently antagonized LPS-induced activation of the NLRP3 inflammasome and inhibition of AMPK in A549 cells. This points to the considerable impact of the AMPK signaling axis on the beneficial effects of SAL in cancer protection and in the context of insulin resistance and atherosclerosis. Notably, Wang et al. [156] recently found that using SAL on A549 cells did not influence the expression level of the EMT marker snail. In contrast, Lee et al. [158] highlighted that farnesol managed to regulate the Akt/mTOR pathway to suppress EMT and hinder tumor progression in a mouse lung cancer model. However, the effects of SAL on Akt/mTOR signaling have displayed distinct outcomes in CRC cells and gastric cancer AGS cells in earlier studies [159,160]. Therefore, further research is needed to ascertain whether SAL indeed influences EMT and what the underlying mechanisms are. Available data emphasize the multifunctional role of SAL in the regulation of cancer by acting on the key signaling pathways, such as p38 MAPK, Akt/MEK/ERK, and AMPK. Such modulation of these pathways would impact processes related to cell proliferation, apoptosis, migration, and invasion; thus, SAL holds great promise in being used for the treatment of different types of cancers. Further research is required to understand its mechanistic role in the regulation of EMT, defining possible context-dependent effects and therapeutic implications in metastatic progression and tumor plasticity. In summary, this study showed that SAL does indeed restrain proliferation and migration in human NSCLC cells through the modulation of the NLRP3 inflammasome, dependent on AMPK signaling.

### 4.5 TCM in lung cancer and drug resistance

Curcumin and berberine are prominent compounds within TCM that have been extensively investigated as potential radiosensitizers [161]. Oidonin, a diterpenoid primarily sourced from *Rabdosia rubescen*s (*Isodon rubescens*) and other plants of the Isodon group, has emerged as a relatively recent contender with radiosensitizing potential within TCM [161]. Oidonin has been reported to have strong anticancer effects in acute leukemia, prostate cancer, breast cancer, and many other kinds of cancerous cells [162,163]. Especially, oridonin has been reported to inhibit the proliferation of lung cancer cell lines and in animal models [164,165]. However, the effect of oridonin combined with radiation on lung cancer cells has not been reported.

An ideal radiosensitizer enhances the ability of radiation to target cancer cells while preserving the radiation sensitivity [166]. In *in vitro* cancer models, the growth of lung cancer cells was effectively hindered by oridonin in a dose- and time-dependent manner, displaying antitumor efficacy both before and following irradiation. Across various radiation dose levels, oridonin heightens lung cancer cells’ radiosensitivity and triggers apoptosis by elevating Bax expression and suppressing Bcl-2 [167]. Specifically, the Bax/Bcl-2 ratio was significantly elevated when oridonin was administered before irradiation, indicating that the combined treatment of oridonin and radiation surpasses the outcomes of each therapy alone in terms of promoting apoptosis [167]. Active Bax recruitment into mitochondria leads to the release of cytochrome c and the formation of pores [168]. In a study conducted by Park et al. [169], oridonin exhibited potent anticancer effects by impeding cancer cells’ growth and clonogenic potential. However, this research also revealed cytotoxicity toward non-cancerous cells, an aspect overlooked in earlier investigations. Notably, oridonin displayed cytotoxicity toward both lung cancer cells and healthy lung epithelial cells. To mitigate adverse effects, oridonin was administered at a lower dose (5 M), reducing its anticancer efficacy compared to previous trials. Nonetheless, the combination of 5 M oridonin with radiation significantly amplified the generation of ROS, DNA damage, and apoptosis in H460 cells. These findings underline the importance of cautious dose adjustments to balance treatment effectiveness and minimize unwanted effects [169]. Oridonin also intervenes in EMT and migration by upregulating E-cadherin and downregulating components like vimentin, snail, and slug [170] and combining oridonin with radiation-enhanced tumor prevention in an *in vivo* model after 7 days of treatment, surpassing the outcomes of administering the two agents separately. This combined approach was associated with increased levels of caspase-3, -H2AX, DNA damage markers, and apoptosis [169]. Taken together, these findings suggest that oridonin has potential as a therapeutic agent, especially in combination with radiation therapy, since it promotes oxidative stress and DNA damage while antagonizing EMT. However, optimization of dosage is important to achieve maximal efficacy with minimal cytotoxicity to normal cells. It requires further investigation in order to better define the combination strategies and explore more extensive clinical applications in the treatment of lung cancer.

In TCM, Tripterygium has an extensive history of addressing diverse medical conditions. Initially employed primarily for rheumatoid arthritis treatment, contemporary research continues to explore its multifaceted attributes [171]. Among the potent components extracted from Tripterygium, quinine methide triterpenoid-Celastrol stands out. This substance profoundly affects both chemotherapy- and radiotherapy-resistant cancer cells, heightening their responsiveness to these treatments through various mechanisms [171]. Conjoining celastrol with radiation augmented the vulnerability of cancer cells to radiation by curbing proliferation and tumor formation, as observed *in vitro* studies involving radioresistant lung cancer cells. Key factors inducing radiosensitivity were pinpointed, including EGFR, ErbB2, survivin, and Akt [161]. Acting as an Hsp90 inhibitor, celastrol significantly lowered EGFR, ErbB2, and survivin levels while sustaining Akt levels. Additionally, it bolstered P53, facilitated the release of mitochondrial cytochrome c, and triggered caspase cleavage (caspase-3, caspase-8, caspase-9, and PARP), ultimately leading to apoptosis [161]. Celastrol heightened ROS during IR, concurrently diminishing the activity of thiol proteins combating free radicals, such as glutathione and thioredoxin reductase.

*Salvia miltiorrhiza*, known as Danshen, is a prominent Chinese herbal remedy. Within this plant lies tanshinone, a phenanthrenequinone compound [172]. Various tanshinone chemicals, classified as anti-tumor agents, exhibit diverse pharmacological actions. Multiple malignancies, including lung, prostate, leukemia, and liver cancers, demonstrated encouraging outcomes in studies [172]. However, the understanding of tanshinone’s effects and mechanisms against radiation resistance in lung cancer remained limited until recently. A recent study by Yan et al. [173] unraveled that the natural compound Tan I sensitized radioresistant lung cancer cells to ionizing radiation (IR). By directly binding to the phosphoribosyl pyrophosphate aminotransferase (PPAT) active site of the protein, Tan I substantially curtailed the production of PPAT, thereby mitigating the PPAT-mediated carcinogenic response in radioresistant lung cancer cells (H358-IR and H157-IR). These findings thus demonstrate that Tan I is an effective adjuvant agent to radiosensitize the resistance of lung cancer and could mean that tanshinone compounds become important adjuvants to enhance the efficacy of radiation therapy for resistant tumors.

*Zanthoxylum schinifolium*, a fragrant plant prevalent in China, Japan, and Korea, houses schinifoline, a 4-quinone alkaloid [174]. Historically used as a spice and recognized for its multifaceted pharmacological properties, *Zanthoxylum* has lately gained attention for its anticancer attributes [175]. Investigating schinifoline’s impact on NSCLC subjected to IR, Wang et al. [174] demonstrated its pro-cytotoxic effects on A549 cells, either alone or in combination with IR, inhibited cell growth. In addition, the study had shown a radiosensitizing effect when cancer cells had been treated with various fraction dosages of IR. Combined with high IR doses, schinifoline markedly reduced the percent of surviving fractions. Notably, pretreatment of A549 cells with schinifoline followed by radiation exposure induced apoptosis, as confirmed by the results of flow cytometry [174]. These results indicate that schinifoline might be a potential potent radiosensitizer in the treatment of NSCLC through promoting apoptosis and reducing cell survival.

Huaier is a fungus that has been reported to have broad anticancer activities against various malignancies, including NSCLC. New research proves that it could inhibit cisplatin resistance in NSCLC through some important signal pathways modulation. In a study by Jin et al. [74], Huaier was found to inhibit the JNK/JUN/IL-8 signaling axis, a critical pathway in cancer cell survival and drug resistance. Mechanistically, Huaier inhibits interleukin-8 (IL-8) expression through the inhibition of nuclear factor kappa-light-chain-enhancer of activated B cells (NF-κB) and activator protein-1 (AP-1), thus attenuating cisplatin resistance and cancer stemness both *in vitro* and *in vivo* [74]. Further investigation showed that a major bioactive constituent of Huaier is kaempferol, which was found to be a powerful inhibitor of resistance to cisplatin. Kaempferol inhibits the activity of c-Jun N-terminal protein kinase (JNK) and subsequently blocks c-Jun phosphorylation and nuclear translocation. This blockage finally reduces the activity of AP-1 that downregulates IL-8 expression, thus increasing cisplatin sensitivity [74].

Another compound derived from TCM, β-sitosterol, also shows promise for overcoming anlotinib resistance in NSCLC. Although anlotinib, a multitarget tyrosine kinase inhibitor, is effective in the treatment of NSCLC, resistance to anlotinib limits its long-term benefit. Recent findings have demonstrated that β-sitosterol may overcome anlotinib resistance through the modulation of the miR-181a-3p/SHQ1 signaling pathway in NSCLC cells. Wang et al. [75] demonstrated that β-sitosterol suppressed miR-181a-3p expression and subsequently increased SHQ1, a key factor of ribonucleoprotein assembly. The latter then downregulated ER stress-related proteins ATF6 and GRP78, thereby inducing apoptosis and inhibiting cell proliferation in anlotinib-resistant NSCLC cells [75]. These findings are an indication that β-sitosterol is an adjunct for overcoming resistance to targeted therapy in lung cancer. In summary, these findings suggest TCM as an adjuvant therapy for overcoming drug resistance in lung cancer. Nevertheless, it is still imperative to focus future research on identifying bioactive compounds, elucidating molecular mechanisms, and conducting rigorous clinical trials with a view toward establishing evidence-based application in lung cancer.

## 5 TCM as a nano-formulation in lung cancer

Nanotechnology-based drug delivery systems seem to be the most promising way to overcome all the limitations linked with conventional therapies in lung cancer [176]. The nanoparticles-based pharmaceuticals exhibit better pharmacokinetics, improved tumor targetability, and reduced systemic toxicity, thus becoming one of the milestones in modern medicine [177]. Particularly noteworthy are nanomedicines utilizing lipid nanoparticles or liposomes, which have become the predominant type of medication authorized by the FDA in the United States [178]. The pioneering nano-drug, Doxil, enclosed within liposomes, received FDA approval. Liposomes can encapsulate both hydrophilic and hydrophobic TCM components owing to their lipid bilayer structure resembling biological membranes and their aqueous inner core [178]. TCM remedies, known for their multi-component and multi-target nature, have demonstrated remarkable clinical efficacy against a wide spectrum of chronic ailments such as pain, liver fibrosis, myocardial ischemia, coronary heart disease, and gastric ulcers [152]. However, their efficacy in treating malignant tumors has been limited. Despite extensive research, active TCM ingredients’ considerable hydrophilicity or hydrophobicity results in rapid elimination and poor absorption. Moreover, the intrinsic instability, limited permeability, and inadvertent off-target toxicity of TCM constituents pose challenges. Investigations are underway to explore the use of liposomes for delivering TCM elements in cancer therapy [179]. Integrating PEG surface modifications and platelet membrane coatings has yielded promising outcomes in liposome-based nanomedicine. Augmented tumor selectivity is a typical outcome of incorporating minor compounds, peptides, antibodies, cell membranes, or magnetic nanoparticles into liposomes [180].

Various active TCM components, including ursolic acid (UA), berberine, and ginsenoside, have exhibited potential intervention effects against lung cancer. Their stability and therapeutic efficiency have been enhanced through self-assembly into nanoparticles (NPs) [181]. UA NPs, for instance, exhibit improved CD4^+^ T-cell activation, substantial inhibition of proliferation, induction of apoptosis in A549 human lung adenocarcinoma cells, decreased expression of COX-2/VEGFR2/VEGFA, heightened immunostimulatory activities of TNF-, IL-6, and IFN-, and reduced STAT-3 activity [182]. UA NPs hold promise for tumor suppression, liver protection, and immunotherapy. PTX-ss-BBR NPs induce G2/M phase arrest, elevate ROS levels, induce apoptosis, and curb tumor growth in A549 cells [183]. Notably, PTX-ss-BBR NPs exhibit enhanced efficacy against *Staphylococcus aureus*, which has been associated with heightened lung cancer risk. Overcoming PTX’s low bioavailability primarily due to its hydrophobicity, self-assembled nanoformulations appear to provide a solution. Through the synthesis of PTX-ss-PTX and subsequent loading with 1,1-dioctadecyl-3,3,3-tetramethylindotricarbocyanine iodide, DiR-loaded self-assembled NPs (DSNs) have been created by Han et al. [184]. The redox-responsive disulfide bond in the disulfide-linked self-assembled nanoparticles (DSNs) allows for rapid and tumor-specific (PTX release, which significantly enhances its therapeutic efficacy. *In vitro* and *in vivo* experiments demonstrate that DSNs exert effective antitumor activity, especially when combined with chemotherapy and photothermal therapy approaches [185]. Under the influence of TME factors such as high intracellular glutathione levels, DSNs release drugs in a controlled and site-specific manner, which may enhance the efficacy of treatment with reduced side effects resulting from less systemic toxicity. Major bioactive component of *P. ginseng*, PPD-type, was then encapsulated into Rb1/PPD NPs for exploiting the enhanced permeability and retention (EPR) effect [185]. This nanoparticle-based approach significantly enhances tumor accumulation, resulting in greater anticancer efficacy and a better safety profile. The nanoformulation of Rb1/PPD not only increases bioavailability but also reduces systemic side effects, which may make it a promising candidate for the treatment of lung cancer [185].

In the realm of TCM, Renshen (*P. ginseng* C. A. Mey.) is employed as a Qi-boosting tonic and Yin-nourishing agent. A bioactive compound called (R)-ginsenoside (Rg3), isolated from Renshen, has exhibited the ability to eliminate cancer cells [145]. Li et al. [186] found when administered to hypoxic NSCLC cells, Rg3 induced a concentration- and time-dependent reduction in the expression of p-p65 and p65. Furthermore, the DNA binding of NF-B was diminished in cells treated with a combination of Rg3 and cisplatin compared to hypoxic cells treated with either drug individually. Remarkably, under hypoxic conditions, the group receiving Rg3 + cisplatin displayed the lowest levels of p-p65 and p65 in nuclear protein extracts and the least expression of p-IKK and IKK in total protein extracts [186]. Consequently, Rg3 effectively prevented the activation of the NF-B signaling pathway under hypoxic circumstances, with this inhibition being significantly enhanced in the presence of both Rg3 and cisplatin. The viability of hypoxic NSCLC cells treated with Rg3 + cisplatin was substantially lower than cells treated with either Rg3 or cisplatin alone. Moreover, colony formation was notably reduced in hypoxic NSCLC cells treated with the combination of Rg3 and cisplatin, compared to separate treatments with cisplatin or Rg3. Flow cytometry analysis revealed a heightened overall apoptotic rate in hypoxic cells treated with Rg3 + cisplatin, in contrast to cells treated individually with cisplatin or Rg3. The Rg3 + cisplatin group exhibited elevated levels of pro-apoptotic proteins (caspase-3, -8, -9, and Bax) and apoptosis indicators (PARP), along with reduced expression of anti-apoptotic proteins (Bcl-2 and survivin). Through the isolation and characterization of *P. ginseng* C. A. Mey., Cao devised a distinctive class of nanoparticles called ginseng-derived nanoparticles (GDNPs) that share similarities with extracellular vesicles (EVs). These GDNPs display remarkable stability and biocompatibility [186]. Exposure to GDNPs significantly heightened the expression of M1-related markers, including CD80, CD86, MHC-II, TLR2/4, IL-6, and TNF-, indicating an M1-like polarization of macrophages following treatment. Notably, the M2-like polarization of macrophages was also modulated *in vitro* by GDNPs, leading to increased production of M1-related cytokines and chemokines such as CCL5, IL-6, MCP-1, TNF-, IL-1, and IL-12. Moreover, treatment with GDNP-stimulated macrophages considerably amplified the apoptosis of B16F10 melanoma cells and the expression of caspase-3/7, surpassing the effects observed with control media treatment [186]. In summary, in consideration of Rg3 combined with cisplatin and effects on NF-κB signaling, apoptosis, and macrophage polarization, the study has shown therapeutic potential in NSCLC. Moreover, the formulation of GDNP from *P. ginseng* is a promising strategy for modulating immune responses to enhance the efficacy of cancer therapies, particularly by targeting tumor cells and supporting antitumor immunity.

## 6 TCM, in combination with modern medicine in lung cancer

In recent years, TCM has been increasingly studied in combination with modern medicine in the treatment of lung cancer with the purpose to enhance therapeutic efficacy, reduce side effects, and improve the quality of life [187]. Integration of TCM with modern oncological treatment includes herbal formulations, acupuncture, mind–body approaches, and chemotherapy, radiotherapy, targeted therapy, and immunotherapy. Localized patients respond well to treatment, although relying solely on chemotherapy carries a significant risk of relapse and metastasis [188]. Chemoradiotherapy is the established approach for lung cancer, yet it can lead to adverse effects and sometimes falls short of providing a cure [189]. With a history spanning thousands of years, TCM has assumed a vital role as a complementary therapy for malignancies [190]. Recent years have witnessed rapid advancements in utilizing small-molecule inhibitors targeting pivotal enzymes involved in metabolic reprogramming, often combined with other anti-tumor medications, to combat cancer [191]. Epigallocatechin-3-gallate, a primary bioactive compound found in green tea, has exhibited an 89.3% inhibition rate against FASN, a key enzyme in fatty acid synthesis. C75, a FASN inhibitor diminishing the activity of the rate-limiting enzyme CPT in fatty acid oxidation, effectively suppresses lung cancer xenograft development [192,193]. Additionally, ND-646, an inhibitor of another crucial enzyme ACC in fatty acid synthesis, curbs fatty acid production and tumor growth in lung cancer mice. When coupled with carboplatin, ND-646 manifests an even more potent effect in quelling xenograft tumors [194]. The small-molecule inhibitor JPH203 blocks the AKT/mTOR pathway, leading to the suppression of lung cancer stem cell growth and a reduction in tumor sphere size. Notably, knockdown of the L-type amino acid transporter 1 also causes PD-L1 downregulation in lung cancer cells. Thus, one of the promising strategies targeting lung cancer stem cells is by combining JPH203 with a PD-L1 inhibitor [195]. In summary, TCM, when combined with conventional anti-cancer therapy, may have the potential to hinder cancer cell dissemination by regulating the metabolic reprogramming of tumor cells.

Combination of TCM as an adjuvant therapy with chemotherapy has demonstrated potential benefits for the treatment of lung cancer. Indeed, platinum-based chemotherapy and TCM combinations are among the most common therapies for NSCLC [196]. Investigators compared the results in patients with lung cancer who have received chemotherapy, with or without supplementation of TCM. In a prospective trial, participants were randomly assigned to two groups using a double blind, multicenter design [80]. Findings indicated that symptoms (vomiting, fatigue, discomfort, dry mouth, and diarrhea) and side effects associated with cisplatin/carboplatin were alleviated in early-stage NSCLC patients [81]. In addition, researchers from Taiwan and China have demonstrated the synergistic effects of the Sun-Bai-Pi extract with cisplatin to enhance its killing efficacy in low-dose cancer cells and their fast elimination in lung cancer patients [197].

Injectable Aidi, an adjuvant chemotherapy medication commonly used in TCM, has gained popularity [198]. Synergistic administration of Aidi injection combined with platinum-based chemotherapy for the treatment of NSCLC showed better efficacy compared to the use of either treatment alone [198]. Notably, the combined treatment has resulted in improvements in the important outcome measures of objective response rate, relative disease control rate, survival rate, and overall clinical effectiveness. Furthermore, this approach has resulted in a 36% reduction in severe toxicities [198,199]. The potency of Aidi injection as a therapeutic agent has been significantly heightened, accompanied by a restoration of impaired cellular immunity. Current data indicate that Aidi injection has the potential to modulate tumor immunity, safeguarding NSCLC patients from the adverse effects induced by platinum-based chemotherapy [198,199]. Concurrently, EGFRs, essential proteins that control cell growth, division, and survival, have emerged as essential players [200,201]. EGFR-TKIs, which can block EGFR signaling, are the standard treatment for patients with EGFR mutations and also very effective to inhibit proliferation of tumor cells. However, using EGFR-TKIs long term has potential side effects such as diarrhea and hepatotoxicity [202]. In a large meta-analysis of the biomedical literature, Chinese medicine combined with EGFR-TKIs resulted in vastly better effects in the total response rate, improvement of quality of life, and 1-year survival rate compared to using EGFR-TKIs alone [203]. In conclusion, EGFR-TKI/TCM combination in treating advanced NSCLC could enhance therapeutic effect and diminish the side effect of medicine. This synergetic interplay helps to extend the scale of effectiveness in individuals battling lung cancer using EGFR-TKI treatment. Therefore, the integration of TCM with conventional therapies, including chemotherapy, EGFR-TKIs, and radiation, holds promise for improving treatment outcomes in lung cancer patients. Modulation of metabolic pathways, immune response, and tumor growth is how TCM provides another layer of therapeutic benefit while potentiating standard treatment and reducing its side effects.

## 7 TCM to reduce the radiotherapy side effects

The management of cancer includes a spectrum of approaches, such as surgery, chemotherapy, radiation, targeted therapy, and more [204]. While treatment usually exerts a positive influence on the control of disease progression, sometimes it gives rise to unsettling side effects such as nausea, vomiting, mucositis, and general weakness, which can lower the quality of life for a patient [204]. TCM has been extensively studied for its potential in reducing the side effects of radiotherapy in cancer patients. TCM approaches, such as herbal medicine, acupuncture, and dietary therapy, may help mitigate radiation-induced damage by modulating immune function, decreasing oxidative stress, and promoting tissue repair [205]. In recent years, numerous patients contending with oral mucositis (OM) have turned to TCM to prevent or alleviate radiotherapy-induced OM [206,207]. Many Chinese herbal medicines possess a “clearing heat” attribute, which can potentially counteract the heat toxin accumulation resulting from radiation therapy. This accumulation may otherwise deplete the body’s vital energy (Qi), bodily fluids, and nourishment (yin), making them valuable in treating OM, according to TCM theory [208]. The antioxidative properties within Chinese herbal medicines can curtail the generation of ROS, subsequently mitigating the severity of mucositis [208]. The antioxidative properties within Chinese herbal medicines can curtail the generation of ROS, subsequently mitigating the severity of mucositis [209].

Turmeric and its bioactive constituent, curcumin, possess antioxidant and anti-inflammatory properties [209]. Interestingly, curcumin mouthwash demonstrated better effects in comparison with chlorhexidine mouthwash in promoting early wound healing in adult patients with chemotherapy- and radiotherapy-induced OM [210]. Ginger is a product of the rhizome of *Zingiber officinale* and traditionally has been used as a medicine worldwide for the relief of muscle pains and aches. Its primary constituents, 6-gingerol and 6-shogaol, have been found to mitigate oral ulcerative mucositis and pain induced by 5-fluorouracil by modulation of Na^+^ channels. This plays a pivotal role in the pain-relieving effect associated with ginger [211]. Tocotrienols, naturally occurring compounds found in vegetable oil, have emerged as potential analogs of vitamin E for cancer therapy, sensitizing cancer cells to chemotherapeutic drugs [212]. Gamma-tocotrienol can restrain 5-fluorouracil-induced ROS production in human oral keratinocytes by maintaining the activation of nuclear factor erythroid 2-related factor 2, a redox-sensitive master regulatory transcription factor [213]. Quercetin, a natural flavonoid abundant in common vegetables and herbs, possesses both antioxidative and anti-inflammatory properties. Administering 250 mg quercetin capsules twice daily for 4 weeks has shown the potential to minimize the occurrence of OM in patients undergoing high-dose chemotherapy for blood malignancies [214]. In summary, natural bioactive compounds such as curcumin, ginger, tocotrienols, and quercetin may become promising therapies to reduce side effects of chemotherapy and radiotherapy. Their antioxidant and anti-inflammatory actions could help to manage OM and other complications, which are very precious adjunctive treatments to conventional cancer therapies.

The integration of Chinese and Western medicine has proven successful in NSCLC therapy. It can enhance chemotherapy and radiation sensitivity while diminishing side effects like bone marrow suppression, nausea, and vomiting. Using Chinese herbal formulations with chemotherapy might also mitigate the toxicity of adjuvant chemotherapy [82]. Compared to platinum chemotherapy alone, including astragalus-based Chinese medicine alongside platinum chemotherapy has been observed to reduce adverse responses resulting from platinum chemotherapy, such as neutropenia, nausea, and vomiting. Astragalus herbal formulas tailored to specific syndrome distinctions have exhibited even greater efficacy than standard astragalus oral medication [215]. Gastrointestinal reactions like nausea and vomiting represent typical adverse effects of anti-tumor treatment. Combinations of acupuncture and moxibustion with gradual intravenous injection of tropisetron hydrochloride, along with moxibustion combined with 5-HT receptor antagonists, have displayed the potential to prevent and alleviate vomiting induced by cisplatin treatment in NSCLC [216,217]. Furthermore, research has demonstrated that specific acupuncture points, “Feishu” (BL 13) and “Lingtai” (GV 10), can impact the metabolism and distribution of PTX in NSCLC mice. Acupoint massage on “Neiguan (PC6)” and “Gongsun (SP4)” has been linked to a notable reduction in the severity of nausea and vomiting [218,219]. Independently of tumor tissue type and chemical composition, applying bioenergy to PC6 acupuncture points has significantly alleviated chemotherapy-induced vomiting symptoms in 70% of patients [220]. Electroacupuncture has also shown the potential to bolster the immunological function of chemotherapy patients [221,222]. Additionally, NSCLC patients who underwent acupuncture treatment experienced a reduced incidence of radiation pneumonia following radiotherapy [220]. In summary, the combination of Chinese and Western medicine is a very effective strategy for the treatment of lung cancer, as it increases the effectiveness of chemotherapy and radiation while reducing toxicity and the side effects usually associated with these treatments. Chinese herbal medicine, acupuncture, and moxibustion give patients better outcomes from treatment, reduced discomfort, and an improved quality of life.

## 8 Limitations and challenges of TCM-based approaches in lung cancer therapy

In the bigger picture, TCM is much safer compared to Western medicine as supported by the cumulative adverse response data in literature [223–225]. However, TCM has to meet the contemporary safety regulations in order to play its role as a modern therapeutic option. TCM substances are derived from nature some of which may pose potential risks to people [225]. The commonly held belief that all TCM remedies are safer than synthesized drugs is a misconception. In reality, any compounds that have effects on the body’s defenses or physiology can cause a broad array of adverse reactions [225]. In other words, the intrinsic toxicities of the TCM treatments are at times underestimated, especially when used as dietary supplements or over extended durations. Numerous Chinese herbs have been recognized to have toxic effects in humans. A comprehensive reference, the Encyclopaedia of Materia Medica, designates 495 out of 5,767 medicinal plants as possessing poisonous attributes [225]. As an example, aristolochic acid obtained from *Aristolochia debilis* Sieb. Et Zucc. (known as Madouling in TCM) has been associated with swift renal failure, illustrated by a cohort of women consuming identical weight-loss supplements [226]. Glycyrrhiza spp. and its derivatives, often employed in TCM for its expectorant, corrective, and harmonizing properties in various formulations, consist of mineralocorticoid components that may result in unfavorable outcomes like edema, hypertension, and disruptions in electrolyte balance [227]. *P. ginseng* C. A. Mey. (known as Renshen or Hongshen in TCM), in the treatment of diverse ailments from simple fatigue and improved circulation, there has been documented an “abuse syndrome.” This syndrome exhibits symptoms such as insomnia, irritability, and increased excitability [228]. To date, the whole effect of TCM on lung cancer cells and its related molecular pathways is not entirely clear. However, TCM acts as an anticancer agent by activating apoptosis and autophagy through pathways such as mTOR and the Bcl-2 family pathway [229]. Certain TCM herbs may even act as radioprotectors, minimizing radiation-induced damage in the normal tissues of cancer patients undergoing radiotherapy. Hence, we should encourage further probing of TCM’s potential in radiosensitization and radioprotection in a clinical setting.

To ensure the future progress of TCM, it is imperative to establish sustainable methods for generating raw materials. The objective should be cultivation following good agricultural practices [230,231]. Moreover, the scientific foundations of daodi merit a more comprehensive investigation. Daodi medicinal materials are planted and produced in specific geographic regions following specified natural conditions and ecological environments. The medicinal materials have specific ways of cultivation, harvesting, and processing, hence becoming popular for their quality and effectiveness compared to the same species of materials from other regions [232]. It is essential to closely monitor the identification and pharmaceutical quality of herbal medical items in both Europe and China, adhering to the criteria set by the pharmacopeia [233]. Considering the complex chemical makeup of TCM medications and the diverse factors contributing to potential differences between batches, traditional quality control measures, similar to those employed for pure chemical drugs, frequently fall short of guaranteeing the quality of TCM substances and formulations.

In order to gain a wider perspective, holistic concepts such as metabolic fingerprinting have to be developed, overcoming the reliance on single-quality marker molecules [234,235]. Funding organizations, publications, academics, and commercial sources must collaborate closely to address this fundamental challenge. Post-harvest treatment and processing (paozhi) hold significant prominence in Chinese herbal medicine. To ensure consistent quality of plant material, these processes require scientific research, the establishment of standard operating procedures, and the definition and implementation of precise endpoints [236]. With new developments in biological, chemical, and computational technologies, interdisciplinary approaches have to be applied to study the evidence-based elements of TCM treatment. Research integrating quality control in the production of TCM products, innovative systems biology, and experience-driven TCM principles will be important for understanding the holistic features of TCM and its eventual incorporation into conventional medicine. To achieve such aims, however, a multidisciplinary approach has to be adopted across the different spheres of expertise. Such cross-cultural research collaboration and open-minded attitude should be encouraged, which will provide a way to foster scientific creativity and studies bridging the gap between TCM practice and the management of well-being and health issues. Sustaining the commitment to integrated scientific approach and practice in research and development is of paramount importance in generating new knowledge to educate the next generation of professionals in the field of integrative medicine, which encompasses TCM practices.

While TCM has shown some potential in the treatment of lung cancer through mechanisms of immunomodulation, induction of apoptosis, and modulation of the TME, there are a number of limitations. Among the major obstacles is that high quality randomized controlled trials (RCTs) are lacking to definitely establish the effectiveness and safety of this intervention [187]. While many *in vitro* and *in vivo* studies provide supporting evidence for the anti-cancer properties of TCM, translation into clinical applications remains scant, with most plagued by small sample sizes, inconsistent methodologies, and poor control groups. Most TCM therapies have not undergone the rigorous multi-phase trials required for modern pharmaceuticals but instead are based on traditional empirical use and observational studies, not standardized, placebo-controlled RCTs. Another major limitation is variability in herbal composition, formulation, and dosage. Unlike single-agent chemotherapy drugs, TCM is often a complex and poly-compound herbal combination, making it difficult to establish standardized dosing regimens with reproducible therapeutic outcomes [237]. Variability may make reproducibility of the results from clinical studies difficult and thus impede regulatory approval under the frameworks of Western medicine. Another important concern with TCM is safety risks and potential regulatory issues. Although TCM is perceived as natural and safe, certain compounds have been reported to induce toxicity, herb–drug interactions, and adverse effects, especially when used concurrently with conventional treatments. For instance, herbs containing aristolochic acid have been associated with nephrotoxicity and carcinogenic effects, leading to regulatory restrictions in many countries [238]. Besides, differences in the control of quality, authentication, and contamination risks between various sources of TCM products call for tighter regulatory control in order to ensure patient safety [239]. Such challenges can be addressed appropriately by future research that focuses on large-scale, well-designed RCTs in order to acquire sound clinical evidence supporting the role of TCM in the treatment of lung cancer. Standardization of herbal formulations and quality control measures together with pharmacokinetic studies will help to increase the reliability and reproducibility of TCM-based treatments.

## 9 Conclusion

Lung cancer is one of the major health problems in the world, and new strategies are needed to improve the treatment effects. TCM has been focused on its potential to modulate the TME and enhance the therapeutic effects of lung cancer treatment. This review aims to provide a comprehensive summary of different findings that emphasize the promising role of TCM compounds in reshaping the landscape of lung cancer treatment. TCM has been a promising avenue to modulate the TME and reinforce the treatment strategies of lung cancer. The multitargeting aspect of TCM, including herbal medicine, is key to the readjustment of the TME, via the regulation of inflammation, immune responses, and cellular signaling. TCM might enhance the therapeutic efficacy of conventional treatments for lung cancer and attenuate their possible adverse effects through the potentials of natural compounds and the interplay among compounds. In the era of tailored therapy and precision oncology, the holistic principles of TCM offer a new outlook that exceeds the simple targeting of cancer cells. With its ability to deal with the complex network of factors in the TME, the potential of TCM to improve treatment outcomes and quality of life for patients, and to forge ahead toward a more holistic approach in lung cancer management, cannot be overstated. With multidisciplinary collaboration and rigorous scientific validation, the place of TCM in the mainstream care of lung cancer will be integrated. Clinical trials and experimental studies should be conducted to understand the mechanisms through which TCM acts on the TME and to establish its role as an adjuvant therapeutic approach. This will facilitate understanding of the mechanisms of action of TCM and guide its optimal incorporation into personalized treatment regimens for lung cancer. Finally, how TCM might modulate the TME is an enticing avenue for future exploration in the landscape of lung cancer treatment. With the advance of scientific understanding and further research, collaboration between conventional oncology and traditional medical practices may open up new dimensions in the fight against lung cancer and eventually bring improved therapeutic strategies for the enhanced well-being of patients.
